# Exploratory Research on Key Technology of Human-Computer Interactive 2.5-Minute Fast Digital Early Warning for Mild Cognitive Impairment

**DOI:** 10.1155/2022/2495330

**Published:** 2022-03-29

**Authors:** Nan Li, Xiaotong Yang, Wencai Du, Atsushi Ogihara, Siyu Zhou, Xiaowen Ma, Yujia Wang, Shuwu Li, Kai Li

**Affiliations:** ^1^School of Humanities and Management, Zhejiang Chinese Medical University, Hangzhou 310053, China; ^2^School of Medical Technology and Information Engineering, Zhejiang Chinese Medical University, Hangzhou 310053, China; ^3^Zhejiang-Japan Joint Laboratory of Digital Diagnosis and Treatment and Equipment for Major Brain Diseases, Zhejiang Chinese Medical University, Hangzhou 310053, China; ^4^University of Saint Joseph, Estrada Marginal da Ilha Verde 14-17, Macau, China; ^5^Department of Health Sciences and Social Welfare, Faculty of Human Sciences, Waseda University, Tokorozawa, Japan; ^6^School of Public Health, Hangzhou Normal University, Hangzhou, China; ^7^Kaibo Medical Equipment (Hangzhou) Co., Ltd., Hangzhou 310052, China

## Abstract

**Objective:**

As the preclinical stage of Alzheimer's disease (AD), Mild Cognitive Impairment (MCI) is characterized by hidden onset, which is difficult to detect early. Traditional neuropsychological scales are main tools used for assessing MCI. However, due to its strong subjectivity and the influence of many factors such as subjects' educational background, language and hearing ability, and time cost, its accuracy as the standard of early screening is low. Therefore, the purpose of this paper is to propose a new key technology of fast digital early warning for MCI based on eye movement objective data analysis. *Methodology*. Firstly, four exploratory indexes (test durations, correlation degree, lengths of gaze trajectory, and drift rate) of MCI early warning are determined based on the relevant literature research and semistructured expert interview; secondly, the eye movement state is captured based on the eye tracker to realize the data extraction of four exploratory indexes. On this basis, the human-computer interactive 2.5-minute fast digital early warning paradigm for MCI is designed; thirdly, the rationality of the four early warning indexes proposed in this paper and their early warning effectiveness on MCI are verified.

**Results:**

Through the small sample test of human-computer interactive 2.5 fast digital early warning paradigm for MCI conducted by 32 elderly people aged 70–90 in a medical institution in Hangzhou, the two indexes of “correlation degree” and “drift rate” with statistical differences are selected. The experiment results show that AUC of this MCI early warning paradigm is 0.824.

**Conclusion:**

The key technology of human-computer interactive 2.5 fast digital early warning for MCI proposed in this paper overcomes the limitations of the existing MCI early warning tools, such as low objectification level, high dependence on professional doctors, long test time, requiring high educational level, and so on. The experiment results show that the early warning technology, as a new generation of objective and effective digital early warning tool, can realize 2.5-minute fast and high-precision preliminary screening and early warning for MCI in the elderly.

## 1. Introduction

Mild cognitive impairment (MCI) is an unstable clinical transition state between normal age and dementia. The study found that people with MCI above 70 have a high prevalence rate, and 21.8% of them progress to dementia 3∼5 years after the onset of MCI. The possibility of transferring to Alzheimer's disease (AD) in patients with MCI is 2.8 times higher than that in healthy aging [1, 2]. As we all know, Alzheimer's disease is still incurable, whose pathological changes are difficult to reverse and the curative effect is poor in the middle and late stage, bringing psychological, physiological, and economic pressure to the patients and their families [3]. However, studies have found that if patients are identified early and reasonably intervened in MCI stage, the reversal rate of mild cognitive impairment to normal cognitive function is more than 30% [4]. Therefore, moving the MCI early warning window forward at the daily home level based on the key technology of human-computer interactive 2.5-minute fast digital early warning is of great significance to improve the life quality of patients and reduce the social burden.

According to the diagnostic guidelines of MCI, its clinical diagnosis is mainly based on medical history collection and neuropsychological scale assessment [5]. Although the neuropsychological scales commonly used in clinic have good reliability and wide application in screening cognitive impairment, some defects are still widespread, such as strong subjectivity, long time consumption, insufficient sensitivity for MCI early warning, and so on [6]. Thus, in order to make up for the deficiencies of cognitive screening scales, more and more computerized cognitive assessment tools [7, 8] appear, but most of them need professionals to carry out the standardized operation, and the subjects need to go to the clinic or examination centers, which immensely limit the universality of detection.

Moreover, the latest research found that, compared with other cognitive fields, the earliest occurrence of cognitive impairment in MCI patients is the decline of visuospatial function, and at present, many studies have used eye movement related examinations to assess early cognitive impairment [6, 9, 10]. At the same time, the powerful computing and data processing ability of intelligent algorithm has its unique advantages in the analysis of eye movement examination results and eye movement trajectory. Many studies have shown that task assessment saccade mode based on eye tracking can be used to detect MCI and early signs of cognitive decline. Detection indexes and forms vary from research to research [11, 12], but all show that task paradigm can be designed to check various visual features of saccade or eyes under relevant stimuli, and eye tracker can be used for activity monitoring to realize extensive and convenient cognitive function examinations.

Based on the summary of previous studies, it can be found that the current tools for MCI early warning and screening have various shortcomings at different levels. Most of scales and questionnaires which are the most commonly used have deficiencies such as long time-consuming and lack of objective assessment data, while the computerized cognitive assessment tools developed in the early stage also have the characteristics such as various inspection types and parameters and complex calculation process and large population variability.

Thus, taking advantage of noninvasive, short time consumption, low cost, and high feasibility of eye movement tracking data acquisition, this paper puts forward four exploratory MCI early warning indexes including “test duration,” “lengths of gaze trajectory,” “correlation degree,” and “drift rate” based on the review and summary of relevant research literature and semistructured expert interviews, in visuospatial function and dynamic eye movement monitoring perspectives.

At the same time, based on the key technology of digital early warning, a human-computer interactive 2.5-minute fast digital early warning paradigm for MCI is designed. Finally, the elderly aged 70–90 with clinical diagnosis conclusion of MCI are included in the subjects, and the human-computer interactive 2.5-minute fast digital early warning paradigm designed in this paper is benchmarked with the clinical diagnosis conclusion, so as to verify the effectiveness of the new digital early warning paradigm in this study.

The new early warning key technology studied in this paper has the advantages of short time consumption and simple operation together with objective and accurate assessment, which can realize the real-time feedback of eye movement trajectory and the accurate preliminary early warning for MCI in the elderly population in 2.5 minutes.

The rest of the paper is organized as follows. In Section 2, this paper introduces the related work on MCI early warning in the literature. In Section 3, this paper explains the research methods of this paper. And this paper introduces the relevant work of the experiment, including the paradigm and principle of experiment, the key technologies of digital early warning, and basic information of experiment in Section 4. In Section 5, this paper analyzes the data processing results. A short discussion of the proposed scheme is presented in Section 6.

## 2. Related Work

By reviewing the literature on MCI early warning methods in Pubmed, Web of Science, and other literature libraries in recent 10 years, we find that, compared with other cognitive fields, visuospatial dysfunction may be the earliest cognitive impairment field in MCI patients [13, 14]. A research [15] showed that there is no significant difference between AD mice and normal control mice when performing olfactory tasks, but on the contrary, the visuospatial function including percentage of visuospatial correct decisions and speed of visuospatial relearning of AD mice was significantly worse than that of control mice, which also confirmed that the decline of visuospatial function may be used as an early sensitive index for MCI screening. A study [16] shows that the scores of spatial structure and connection test of MCI patients are low, speculating that visual reasoning, connection, and spatial structure test may be sensitive to the early diagnosis of MCI patients, or the cognitive function reflected by such test, that is, visuospatial and executive function, has diagnostic effect for MCI early diagnosis. In addition, some pathologists [17] found that the initial lesion location of numerous patients with MCI is not the well-known hippocampal region, but the visual contact cortex, which also shows that the loss of visuospatial function may occur before the symptoms of hippocampal involvement (such as memory decline).

At present, many studies have used examinations related to eye movement to assess early cognitive impairment. Study of Bylsma has found that frequency of saccadic intrusion of patients with cognitive impairment may increase due to the decline of attention and working memory function, which is negatively correlated with MMSE scores [18]. VisMET, designed and developed by Haque et al. [19], can assess the visuospatial memory of healthy aging and mild cognitive impairment by analyzing the subjects' gaze points and gaze duration, so as to realize assessment for MCI.

With the continuous development of modern medicine, there are more and more types of computerized cognitive screening systems. Bartoli et al. [20] used Omni robot to control and move the patient's arm to complete a test and analyzed the visuospatial ability of MCI patients through the recorded trajectory and eye movement data. Nevertheless, this study analyzed many variables and needs long time to complete, so it is difficult for the elderly. A computerized cognitive screening test developed by Canadian researchers can screen cognitive impairment through three cognitive domains: information processing speed, working memory, and executive function [21], but it takes 10 minutes and lacks scalability for rapid screening of large-scale populations. Besides, Papp et al. team [22] developed a long-term episodic memory management and measurement method suitable for those at risk of AD by using image stimulation from the perspective of episodic memory. Subjects can detect episodic memory ability through a mobile application, but it mainly assesses cognitive ability from the memory dimension, with low efficiency of MCI early screening and warning.

Consequently, although the existing cognitive screening information system has many advantages, the screening for MCI patients still has the following deficiencies:

1. Systematic screening needs professional doctors. At present, it is difficult to carry out large-scale community screening under the current situation of lacking doctor resources.
2. The system test is highly difficult and time is too long, so it is not suitable for fast primary screening in large-area home crowd.
3. The design of test paradigm is not objective enough, the degree of standardization is low, and there is a lack of objective and quantitative assessment indexes. The early warning effectiveness is not enough, and it is difficult to meet the needs of screening.
4. There are still few studies on digital early warning screening for detecting MCI from the perspective of visuospatial dysfunction.

See Table 1 for more detailed analysis of deficiencies, which also shows the research gaps found in previous studies in the field of MCI early warning.

At the same time, we found that numerous studies assess early cognitive impairment through eye movement data, indicating that eye movement test is a relatively simple and objective assessment and screening tool for MCI at present. Although detection indexes and forms vary from research to research, the assessment of eye movement test in these studies often focuses on test durations, eye drift, and so on. Based on this, aiming at the shortcomings of MCI early warning at present, a human-computer interactive digital early warning paradigm for MCI is designed based on visuospatial function assessment and dynamic eye movement monitoring. It realizes eye movement dynamic follow-up and real-time feedback under time sequence and outputs visual dynamic analysis results in time on the basis of the concept of scene activation. Concurrently, the fast, accurate, and digital early warning and screening for MCI high-risk population based on eye movement objective data analysis is realized.

## 3. Methodology

### 3.1. Semistructured Interview

In order to get more reliable information, we use semistructured interview to determine the effective early warning indexes for MCI. The 20 experts participating in the interview are composed of neurologists, interdisciplinary scholars of neurocomputing, and elderly health management experts, who all have rich experience in the assessment and diagnosis of MCI in large general hospitals.

Based on previous literature review [25–28], this paper formulates an interview outline. By designing the outline of behavioural event interview, the interviewees are reviewed in an open-ended manner so as to dig out the indexes related to MCI early warning. Three days before the formal interview, the interview outline was provided for the interviewees for preparation, so as to improve the effect and quality of the interview. In addition, before the formal implementation of the interview, this paper clearly informed the interview process and the purpose of the obtained data, so as to avoid the interviewees' worries. The outline of the interview is shown in Table 2.

### 3.2. Key Technology of Eye Movement Objective Data Acquisition

**Figure 1:**
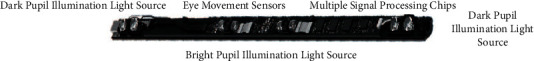
Internal structure of eye tracker.

The objective data acquisition of eye movement is realized by Tobii eye tracker, composed of two eye movement sensors, dark pupil illumination light sources, bright pupil illumination light source, and multiple signal processing chips, whose internal structure is shown in Figure 1.

**Figure 2:**
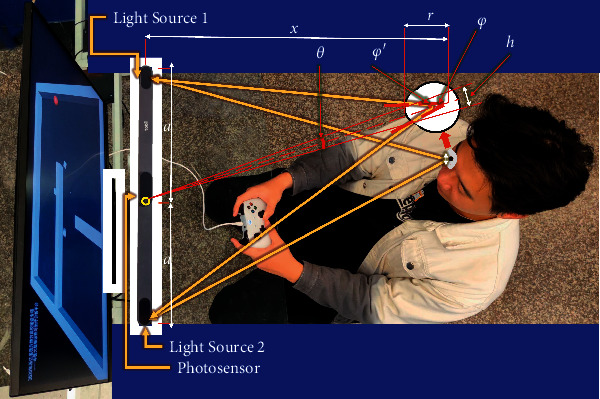
Light source reflection diagram.

Tobii eye tracker uses multiple near-infrared light sources as reference points for auxiliary analysis. By collecting the reflected light from the pupil and cornea, the eye tracker analyzes the relative position of eyes and then obtains the focus of the user's line of sight. On the basis of multireference point complementary technology, the user's head trajectory compensation is realized to ensure the accuracy of the collected data.  Figure 2 shows light source reflection diagram. The technology adopted by Tobii eye tracker is an improved version of the traditional pupil center cornea reflection (PCCR) eye tracking technology (US patent us7572008) [29], and the principle is as follows:

(1) $$\begin{array}{c} d∼\theta \\ h\approx x\theta \\ h=2rsin\frac{\varphi }{2}\\ \varphi =\varphi^{\prime }-\frac{\theta }{2}\approx \varphi^{\prime }\\ \varphi^{\prime }\approx arctan\frac{a}{x}\\ d∼\frac{r}{x}sin\left[arctan\left(\frac{a}{x}\right)\right] \end{array}$$

**Figure 3:**
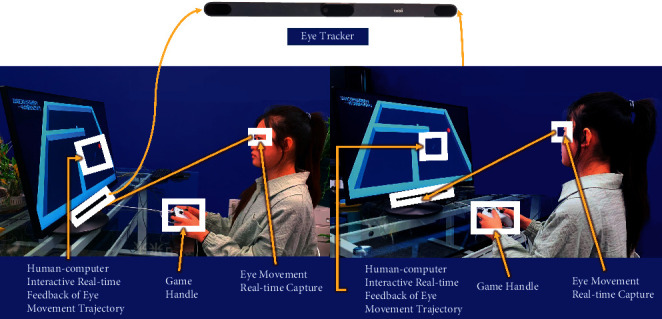
The principle of human-computer interactive eye tracking.

Based on the principle shown in Figure 3, the human-computer interactive 2.5-minute fast digital early warning paradigm for MCI studied in this paper is completed on a computer equipped with eye tracker. Below the computer display is the eye tracker used in this study. In order to explain the calculation method more clearly, we define the position information of the subject's line of sight on the display collected by the eye tracker as “gaze sample,” whose sampling rate studied in this paper is 60 Hz, which is about to collect the subject's “gaze sample” every 0.0167 seconds. The principle of human-computer interactive eye tracking is shown in Figure 3.

## 4. Experimental Design

### 4.1. Experimental Design of Human-Computer Interactive Fast Digital Early Warning Paradigm for MCI

The key technology of early warning proposed in this paper serves the elderly high-risk groups with cognitive impairment and is committed to the early warning and screening of MCI in the home or community scene, which should meet the use needs of high-risk groups with cognitive impairment. First of all, designing digital games for elderly users need to be user-centered, which should be suitable for the knowledge and understanding of elderly players [30]. Therefore, this study is based on the principle of human factors engineering, follows the user-centered design principle, and finally completes the game design based on the preexperiment of multiple schemes in the early stage.

Through the preliminary preexperiment, we found that most of the elderly in the community have varying degrees of vision problems, and most of them are right-handed. Hence, considering the actual situation of users and the reasonable distance of data measurement, this study uses a 32-inch 4K ultraclear display screen, equipped with high-performance miniaturized eye tracker (Tobii eye tracker 5) and Xbox handle as operating device. The eye tracker can be installed under the computer display for eye movement perception and acquisition. During the experiment, each subject needs to sit 55 cm away from the device to meet the requirements of vision and data acquisition.

The subjects complete the test through operating handle, which is easy to move and has a round touch. Besides, the rocker is mainly used in the operation, which is an easier operation method to master, compared with the keyboard and mouse. It is simple to use and can enhance the sense of main control and have a high degree of adaptability to the aging.

**Figure 4:**
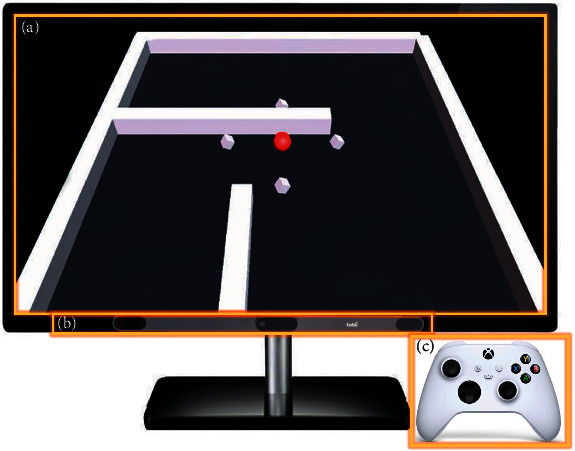
Overall presentation. (a) Paradigm interface of red ball. (b) Eye tracker. (c) Handle.

In terms of paradigm design, the interface designed in this study is mainly black and white, and the object controlled by the subjects is a red ball. The color contrast between red ball and the interface is strong, making the target prominent. As for designing obstacles and eliminating targets, the subjects are mostly right-handed operation, so the main obstacles are set on the left side of the interface to facilitate the subjects to operate in a relatively fixed posture. Meanwhile, considering that the elderly have obstacles in understanding that the targets need to be removed from the paradigm, the four targets in the interface are set to dynamic 3D rotation. See Figure 4 for overall presentation.

**Figure 5:**
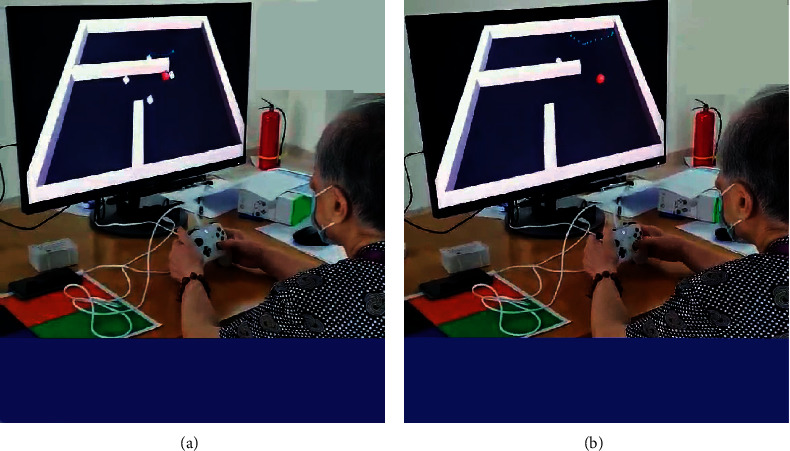
A case of experiment. (a) The scene of the test starting. (b) The scene of the test going to end.

Through the preexperiment of 80 samples in the early stage, it is found that the total time for most people with normal cognition to complete the paradigm will not exceed 2.5 minutes (150 seconds). Thus, when designing the paradigm, 2.5 minutes will be taken as cut-off point of the reference paradigm time. Subjects who still fail to complete the paradigm after 2.5 minutes are considered to have a high risk of MCI.  Figure 5 shows a case of experiment.

**Figure 6:**
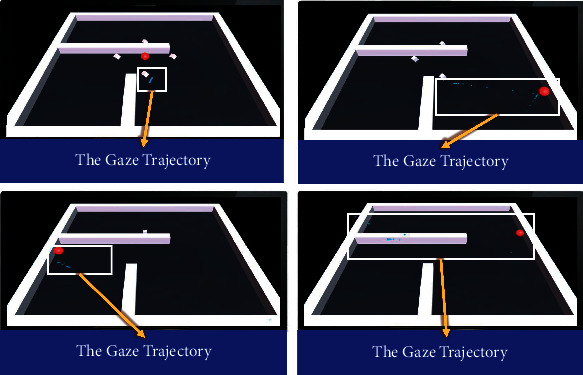
Test paradigm for the purpose of eliminating cubes.

The paradigm we designed consists of three modules: information entry, white cube elimination, and data feedback. In order to ensure that the equipment can accurately detect the subject's eyes, the equipment needs to be calibrated before the test. The test rules and the method of operating the equipment will be orally informed to the subjects by professionals. In the module of information entry, the staff can click the start button to conduct the formal test after entering the subject's name. In the module of white cube elimination, subjects need to use the rocker of the Xbox handle to control the movement of the red ball to collide and eliminate four white cubes. The test paradigm is shown in Figure 6.

**Figure 7:**
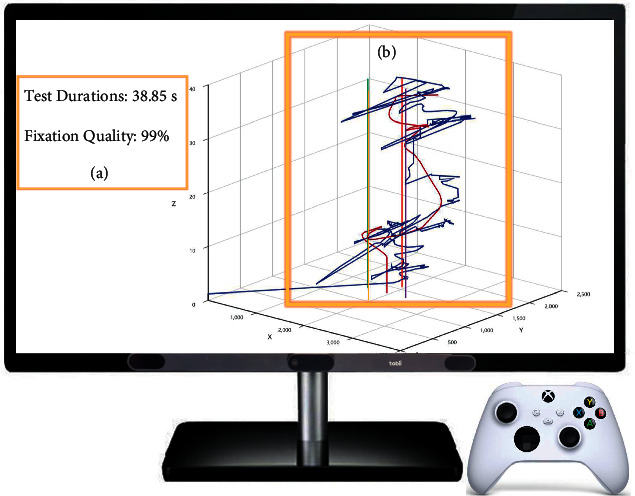
Data feedback. (a) Test durations. (b) Gaze trajectory and ball trajectory.

After the four white cubes are all eliminated, the subjects can receive their test durations in the module of data feedback on the screen. What is more, the eye movement trajectory will be displayed also. Both the test data are fed back to the user as soon as the test ends, as shown in Figure 7.

### 4.2. Experiment Implementation

#### 4.2.1. Data Acquisition and Principle in Test Paradigm

**Figure 8:**
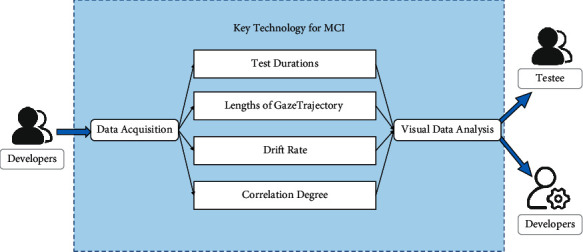
Key technology of human-computer interactive fast digital early warning for MCI.

Based on the summary of relevant literature and semistructured expert interviews, we dug out four exploratory MCI early warning indexes including test durations, lengths of gaze trajectory, correlation degree, and drift rate. The digital paradigm and mathematical analysis technology are applied to assess the visuospatial function and dynamically monitor the eye movement, so as to realize the preliminary screening and early warning for MCI. This technology is mainly realized by designing the paradigm scene in which the subjects control the red ball to eliminate the four white cubes in the virtual environment through collision. Finally, the test durations and the dual trajectories graph of gaze and read ball will be fed back to users. The framework of key technology of human-computer interactive fast digital early warning for MCI is shown in Figure 8.

**Figure 9:**
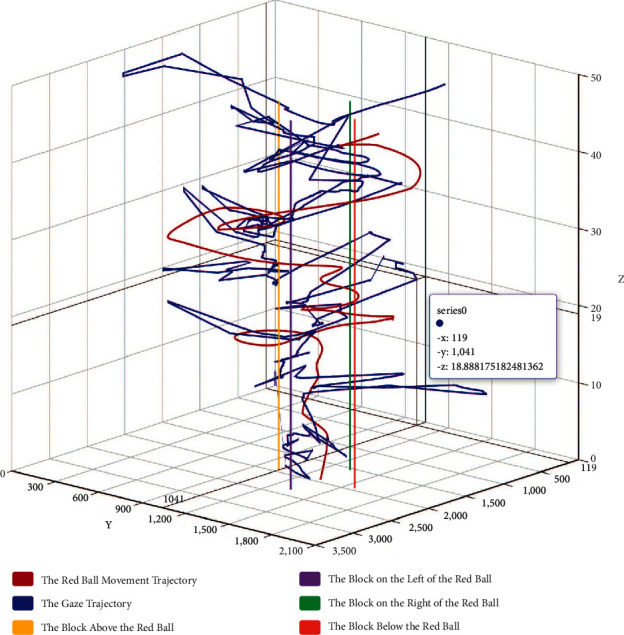
A case of 3D dual trajectories graph.

Moreover, in order to observe and analyze data more accurately and intuitively, Ecarts is used to visualize the data in this study. As shown in Figure 9, in the 3D coordinate diagram, the *X* axis represents the abscissa of the red ball movement trajectory and gaze trajectory, the *Y* axis represents the ordinate of the red ball movement trajectory and gaze trajectory, and the *Z* axis represents the time of the paradigm. Then, the gaze sample position, red ball position, and four white cube positions in the paradigm are represented in the figure with different color labels, respectively. In the graph, dark red curve represents the red ball movement trajectory and navy-blue curve represents the gaze sample trajectory. Purple, yellow, green, and red lines, respectively, to represent the positions of four white cubes.

**Figure 10:**
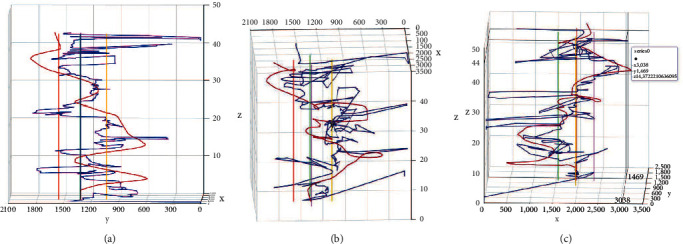
3D dual trajectories graph from different angels. (a), (b), (c) 3D dual trajectories graph from different angels.

Subjects can obtain the visualization results of paradigm data after the paradigm ends and retain the corresponding trajectory by checking different labels. 3D dual trajectories graph from different angels is shown in Figure 10. Nontechnical personnel can make an intuitive comparison by observing the similarity between the gaze trajectory and the red ball trajectory and then get a general judgment, while professional data analysis needs further calculation.

#### 4.2.2. Definition and Quantitative Analysis of Key Indexes in Test Paradigm

Based on the previous literature review and semistructured interviews, this study identified four key indexes including test durations, lengths of gaze trajectory, correlation degree, and drift rate. In this paradigm, the total time is *T*(s), the sampling time of the gaze samples and the position of the red ball in *T* is *I*, the time point of single sampling is *t*_*i*_, the single gaze sample position is *Gaze*_*i*_, and the single position of red ball is *Ball*_*i*_(0 < *i* ≤ *I*, *i* ∈ *N*^*∗*^).

*L*_*Gaze*_ means length of gaze trajectory in this paradigm, which is the total length of the gaze sample trajectory. The calculation method is as follows:

Make *d*_*i*_ the distance between two adjacent gaze samples:

(2) $$\begin{array}{c} d_{i}=\sqrt{{\left({\text{X}}_{Gaze_{i+1}}-{\text{X}}_{Gaze_{i}}\right)}^{2}+{\left({\text{Y}}_{Gaze_{i+1}}-{\text{Y}}_{Gaze_{i}}\right)}^{2}}\left(1\leq i\leq I-1,i\in N^{*}\right) \end{array}$$

where *X*_*Gaze*_*i*+1__  refers to the abscissa of the gaze point at the *i* + 1th sampling, and *X*_*Gaze*_*i*__ refers to the abscissa of the gaze sample at the *i*th sampling; Y_*Gaze*_*i*+1__ refers to the ordinate of the gaze sample at the *i* + 1th sampling, and Y_Gaze_i__ refers to the ordinate of the gaze sample at the ith sampling.

Lengths of gaze trajectory *L*_*Gaze*_ can be obtained:

(3) $$\begin{array}{c} L_{Gaze}=\sum_{i=1}^{i=I-1}d_{i}. \end{array}$$

In this paradigm, the correlation degree is used to describe the similarity between the red ball trajectory and the gaze trajectory. The algorithm of the correlation degree is based on the correlation coefficient, which is the quantity of the linear correlation degree between the research variables. The formula is as follows:

(4) $$\begin{array}{c} corr\left(a,b\right)=\frac{cov\left(a,b\right)}{\sqrt{var\left[a\right]\times var\left[b\right]}}. \end{array}$$

*cov*(*a*, *b*) is the covariance between *a* and *b*, *var*[*a*] is the variance of a, and *var*[*b*] is the variance of *B*.

Because the red ball trajectory (the red ball coordinate position sequence, *Ball*) and the gaze trajectory (the gaze coordinate position sequence, *Gaze*) are not simple one-dimensional random variable data, the abscissa and ordinate of the two sequences are separated to form the corresponding sequence separately. *X*_*Ball*_ is the red ball abscissa sequence, *Y*_*Ball*_ is the red ball ordinate sequence, *X*_*Gaze*_ is the abscissa sequence of gaze samples, and *Y*_*Gaze*_ is the ordinate sequence of gaze samples. At this time, correlation degree of *X*_*Ball*_ *an*  *d* *X*_*Gaze*_, that is,  *X*_*corr*_, and correlation degree of *Y*_*Ball*_ *an*  *d* *Y*_*Gaze*_, that is, *Y*_*corr*_, can be calculated, respectively.

Finally, the correlation degree is obtained:

(5) $$\begin{array}{c} corr=\frac{X_{corr}+Y_{corr}}{2}. \end{array}$$

In this study, Ecarts is used to visualize the test results of MCI early warning paradigm, and the 3D dual trajectories graph of the gaze trajectory and red ball trajectory of the subjects is formed. The experimental legend shows that the trajectory of some subjects shows the sharp angle of gaze trajectory and is far away from the red ball trajectory, which is unreasonable, because the goal of the paradigm designed in this paper is to let the subjects control the red ball to eliminate the white cubes. At the same time, when setting the numerical range, this test allows a certain limit of line-of-sight offset caused by vision, sitting position, and instrument error. Therefore, if the distance between the subject's gaze sample and the red ball position exceeds a certain range at the same time, it is regarded as eye drift.

In this study, the project team explored the value of eye drift threshold between 100 px and 1000 px and took every 50 px as the standard interval, with a total of 21 methods. The Apriori algorithm is used to analyze the correlation between the subjects' gaze sample data under different values and the subjects' final judgment results. The results show that the confidence is the highest at the thresholds of 400 px, 450 px, and 500 px, all reaching 0.89. Furthermore, the project team conducted logistic regression analysis on the three groups of data with the highest confidence. It was found that the prediction effect was the best at the threshold of 450 px.

Drift rate is calculated as follows:

In formula (6), *L*_*Gaze*−*Ball*_*i*__ is the length between the gaze sample and the position of the red ball at the *i*th sampling.

(6) $$\begin{array}{c} L_{Gaze-Ball_{i}}=\sqrt{{\left(X_{Gaze_{i}}-X_{Ball_{i}}\right)}^{2}+{\left(Y_{Gaze_{i}}-Y_{Ball_{i}}\right)}^{2}} \end{array}$$

Formula (6) is a function to judge whether the gaze drifts away. In formula (7), *D* is the number of distances beyond 450(*px*) between the red ball and the gaze sample in all samples. That is, *L*_*Gaze*−*Ball*_ ≥ 450(*px*).

(7) $$\begin{array}{c} f\left(L_{Gaze-Ball_{i}}\right)=\left\{\begin{array}{c} 1\;L_{Gaze-Ball_{i}}\geq 450\left(px\right)\\ 0\;L_{Gaze-Ball_{i}}<450\left(px\right) \end{array}\right.\;\left(i\leq I,i\in N^{*}\right), \end{array}$$

(8) $$\begin{array}{c} D=\sum_{i=1}^{i=I}f\left(L_{Gaze-Ball_{i}}\right)\left(0<i\leq I,i\in N^{*}\right) \end{array}$$

Finally, drift rate is obtained:

(9) $$\begin{array}{c} \text{Drift Rate}=\;\frac{D}{I}\times 100\% \end{array}$$

#### 4.2.3. Participants

MCI patients and the normal cognitive aged in a medical institution in Hangzhou were taken as the research objects; the human-computer interactive 2.5-minute fast digital early warning paradigm test for MCI was carried out. As four early warning indexes of MCI fast digital early warning, test durations, lengths of gaze trajectory, correlation degree, and drift rate were recorded.

Inclusion criteria of MCI group are as follows: (1) at the age of 70–90 years; (2) with a clear clinical MCI diagnosis; (3) able to complete the test independently. Inclusion criteria of normal cognitive group are as follows: (1) cognitive impairment being excluded clinically; (2) with a clear clinical MCI diagnosis; (3) able to complete the test independently;

See Table 3 for exclusion criteria.

#### 4.2.4. Rationality Test of Key Indexes for MCI Early Warning

SPSS 26.0 statistical software is used for data processing. The measurement data conforming to the normal distribution is represented by $\bar{x}\pm s$ and data between two groups are analyzed by independent sample *t*-test. The measurement data not conforming to the normal distribution are represented by the *M* (quartile) and data between two groups are analyzed by nonparametric test. Compare the characteristics of test durations, lengths of gaze trajectory, correlation degree, and drift between MCI group and normal cognitive group (*p* < 0.05). The prediction performance for MCI of each index is analyzed by ROC curve and AUC.

## 5. Results

### 5.1. Results of Semistructured Interview

This study summarizes the interview results, as the following shows.

1. The important goal of constructing MCI early warning paradigm is to detect and screen MCI quickly and effectively, so as to reduce the conversion rate of MCI to AD and reduce the burden on families and society.
2. Eye movement examination is an effective way to detect and assess MCI.
3. The objective early warning indexes for MCI mainly include test duration, lengths of gaze trajectory, correlation degree, and drift rate.
4. The deficiency of current MCI early warning technology is the lack of objective dynamic assessment index and fast assessment mode.
5. It is expected to build a new fast digital early warning model for MCI in the future.

**Figure 11:**
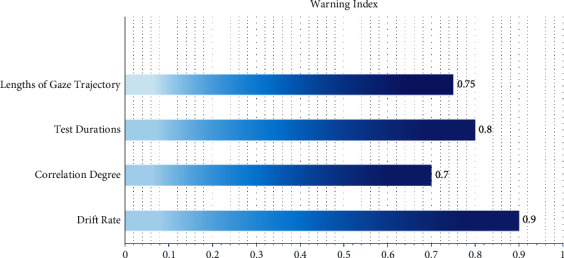
Indexes and coefficient with high frequency base on semistructured interview.

Figure 11 shows indexes and coefficient with high frequency based on semistructured interview.

### 5.2. Results of the Experiment

#### 5.2.1. Participants Characteristics

A total of 36 subjects were collected in this experiment, and 4 cases were excluded due to cataract, stroke, and depression. A total of 32 subjects meeting the above criteria were included, including 16 patients with MCI and 16 normal cognitive aged. There was no significant difference in sex ratio (*p* = 0.373), age (*p* = 0.064), and education level (*p* = 0.175) between the two groups, which eliminated the effect of age, gender, and education level on the follow-up test results. See Table 4 for statistical analysis results.

#### 5.2.2. Comparison of Three Indexes between MCI Group and NC Group

In order to further determine whether the indexes of key technology of human-computer interactive fast digital early warning between MCI group and NC group is discriminative, this paper statistically analyzed the test durations, lengths of gaze trajectory, correlation degree, and drift rate between two groups. The results are shown in Table 5.

Test durations (s) of MCI group are 50.44 (85.89) and those of NC group are 43.39 (42.22). There is no significant difference in test durations between the two groups (*p* = 0.407). Lengths of gaze trajectory (pixels) in MCI group are 11562.63 (9539.73), and those in NC group are 10474.51 (6638.62). There is no significant difference in the distribution of lengths of gaze trajectory between the two groups (*p* = 0.274). Correlation degrees of MCI group are 0.74 ± 0.19, and those of NC group are 0.87 ± 0.08. There is significant difference in correlation degrees between the two groups (*p* = 0.001). Drift rate of MCI group is 0.28 (0.27), and that of NC group is 0.16 (0.18). There is significant difference in drift rate between the two groups (*p* = 0.019). The measured values of correlation degree in MCI group are slightly lower than that of NC group and drift rate of MCI group is greater than that of NC group, and the data between the two groups are statistically significant.

**Figure 12:**
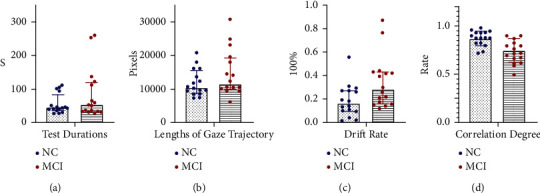
Comparison of indexes between MCI and NC. (a) Distribution of test durations. (b) Distribution of lengths of gaze trajectory. (c) Distribution of drift rate. (d) Distribution of correlation degree.

Figure 12 is drawn by Graphpad Prism 9.0, showing the general distribution of data between the two groups.

#### 5.2.3. Assessment Results of Key Indexes Screening Rate Based on ROC Curve

The prediction performances for MCI early warning of the two indexes (correlation degree and drift rate) in human-computer interactive 2.5-minute fast digital early warning test for MCI are determined using a ROC analysis. An area under the ROC curve (AUC) is used as an index of prediction performance for discriminating MCI from normal cognitive aged.

**Figure 13:**
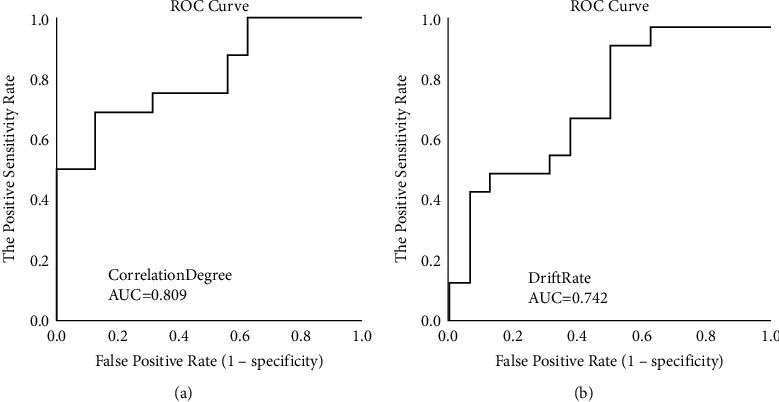
ROC curve of single index for MCI early warning. (a) ROC of correlation degree. (b) ROC of drift rate.

Firstly, we explored the ROC curve and the area under the ROC curve (AUC) for MCI early warning of correlation degree and drift rate, respectively. As shown in Figure 13, AUC of correlation degree is 0.809, and AUC of drift rate is 0.742.

**Figure 14:**
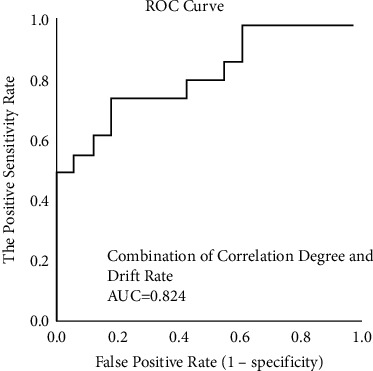
ROC curve of joint indexes for MCI early warning.

In order to further improve the prediction performance for MCI early warning, we paired the two indexes with discriminative significance (correlation degree and drift rate) and operated the ROC curve analysis. As shown in Figure 14, AUC of combining correlation degree and drift rate is 0.824.

## 6. Discussion

Firstly, based on the key technology of human-computer interactive 2.5-minute fast digital early warning, this paper explores the early warning performance of the four indexes of test durations, lengths of gaze trajectory, correlation degree, and drift rate for MCI through the small sample test of 32 subjects aged 70–90. In the preexperiment, we found that the maximum test durations of the normal cognitive population (including the elderly with unskilled operation) in the human-computer interactive early warning paradigm in this paper are 1 minute and 50 seconds. In order to prevent special situations, we have extended it by 40 seconds (for avoiding some special minor situations during the test, so the elderly with the test durations more than 2.5 minutes can end the test and directly determine being at the risk of MCI. After the experiment, it is found that there is no significant difference in test durations and lengths of gaze trajectory between MCI and NC (*p* > 0.05), and there are significant differences in correlation degree and drift rate between the two groups (*p* < 0.05). The results confirm that the human-computer interactive 2.5-minute fast digital early warning paradigm for MCI designed in this paper is not related to test durations in people with normal cognition and early MCI, if test durations are less than 2.5 minutes, which breaks our view that the longer the test durations are, the more serious the MCI condition is (even when the test duration of MCI patients is less than that of people with normal cognition). Nevertheless, the two MCI early warning indexes, correlation degree and drift rate, still show good early warning effectiveness. Hence, within 2.5 minutes of the early warning prediction paradigm, some normal cognitive aged will increase the test durations due to their unskilled operation of electronic devices, but the increase will not affect the two key MCI early warning indexes: correlation degree and drift rate.

Secondly, we analyze the early warning performance of correlation degree and drift rate for MCI, respectively. In the analysis of early warning performance of the single index for MCI, the AUC of correlation degree is 0.809; the AUC of drift rate is 0.742, which is consistent with the research conclusion [31] that saccadic intrusions greatly increase in eye movement dynamic examination in patients with impaired cognition. Based on the above results, we can conclude that, under the mode of early warning performance of the single index, the prediction effectiveness of correlation degree is slightly higher than that of drift rate. Thus, the early warning effectiveness of correlation degree is better.

Subsequently, we further explored the ROC curve of MCI early warning after the combination of correlation degree and drift rate. AUC of combining correlation degree and drift rate is 0824, which is higher than AUC of correlation degree (0.809) and AUC of drift rate (0.742), indicating the MCI prediction performance of combining correlation degree and drift rate is higher than that of single index in the experiment.

**Figure 15:**
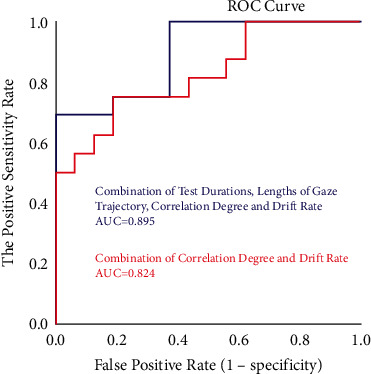
ROC curve of combing two indexes with discriminative significance and two key indexes for MCI early warning.

Next, in order to further explore the prediction performance of MCI early warning indexes, two indexes (correlation degree and drift rate) with discriminative significance and two key indexes (test durations and lengths of gaze trajectory) are combined for joint early warning analysis. Although there is no significant difference in the distribution of test durations and lengths of gaze trajectory between the two groups in part 5.2.2, the experiment results show that the area under the ROC curve (AUC) is improved when the two key indexes are combined with two indexes with discriminative significance as shown in Figure 15. The area under the ROC curve of combining test durations, lengths of gaze trajectory, correlation degree, and drift rate for MCI early warning and prediction is up to 0.895, which is higher than the early warning effect of the combined use of the two digital biomarkers. The occurrence of these situations may be related to the small sample size of this paper.

In conclusion, the key technology of human-computer interactive 2.5-minute fast digital early warning for MCI proposed in this paper (1) realizes the collection of eye movement objective data through eye tracker and achieves the extraction and analysis of eye movement data by using the objective assessment key technology, which overcomes the lack of objective assessment data of screening scales in common use. (2) And by assessing visuospatial function which is an early sensitivity assessment dimension for MCI, it makes up for the defects of low sensitivity and screening rate of screening scales for MCI. Besides, (3) based on the dynamic automatic paradigm scene combined with human-computer interaction, it overcomes the shortcomings of long time consumption and requiring professionals to assess screening scales and previously developed computer systems, which makes it easier for subjects to obtain an activated test experience in the test. Early warning in the elderly population through experimental verification realizes the fast and high-precise MCI screening and early warning in 2.5 minutes among the elderly population and constructs a new model for MCI early warning based on “correlation degree” and “drift rate.”

There are three shortcomings of this paper. (1) The subjects of this study are in the age range of 70–90 years, who are generally older. It is found that the sensitivity is low when carrying out the human-computer interaction paradigm for some people under the age of 60. Therefore, in the later stage, we will develop grouping devices of different ages to automatically adjust the early warning sensitivity according to different ages. (2) In this paper, the subjects only cover MCI and normal cognitive aged, not including AD population. (3) The total sample size of this experiment is limited, and further experiments need to be carried out to expand the universality of the research results. Future research will explore the utility of this technology in the mild cognitive impairment, and AD population of different ages and establish free MCI human-computer interactive early warning program and public case database for all researchers in https://brainhealth.zcmu.edu.cn/en/mciew.

## Figures and Tables

**Table 1:** Previous studies and research gap.

Research	Method	Limitation
P. Maruff, Y. Y Lim, D. Darby, et al. [23]	Novel method for rapid assessment of cognitive impairment using high-performance eye tracking technology	To assess by staring at still images, with long time consumption, not activating the human-computer interactive scene, lacking dynamic follow-up of eye movement in real time under time sequence, not realizing the automatic test function
A. Oyama, S. Takeda, Y. Ito, T. Nakajima, et al. [24]	Through the functional assessment from visuospatial memory ability realizing MCI early warning	
R. U. Haque, C. M. Manzanares, L. N. Brown, et al. [19]	Through the assessment from visuospatial learning ability and memory defect for MCI early warning	

E. Bartoli, F. Caso, G. Magnani, and G. Baud-Bovy [20].	Using robot to control and move the patient's arm to complete a test and analyzing the visuospatial ability of MCI patients through the recorded trajectory and eye movement data	The operation is difficult for the elderly and takes long time

I. E. Plattner, L. Mbakile-Mahlanza, S. Marobela, et al [21]	Screening cognitive impairment through three cognitive domains: information processing speed, working memory, and executive function	Long time consumption

R. F. Buckley, K. P. Sparks, K. V. Papp [7]	Using image stimulation from the perspective of episodic memory for nonclinical AD	The main function is to assist in the diagnosis and judge the progress and severity of AD, and the early warning efficiency of MCI is low

**Table 2:** Interview outlines.

Interview outlines
1. What do you think is the goal of MCI early warning?
2. What do you think is the most effective way to assess MCI?
3. Which objective early warning indexes do you think can realize MCI early warning?
4. What do you think are the shortcomings of the current MCI early warning methods?
5. What are your expectations and suggestions for MCI early warning mode in the future?

**Table 3:** Exclusion criteria.

Serial number	Item
1	Tumour
2	Uncontrollable diabetes
3	Brain trauma
4	History of drug abuse
5	Known learning disabilities
7	Apoplexy
8	Serious medical diseases
9	Depression
10	Nonprimary cognitive impairment
11	Significant visual impairment

**Table 4:** Participant characteristics between MCI group and normal cognitive (NC) group.

Items	Classification	Total population	MCI group	NC group	*p* value
Gender (*n*, %)	Male	6 (18.75%)	2 (33.33%)	4 (66.67%)	0.373
	Female	26 (81.25%)	14 (53.85%)	12 (46.15%)	
Age (years)	84.50 (7.00)		86.00 (3.00)	83.00 (10.75)	0.064
Educational level (years)	12.00(6.75		12.00 (6.00)	9.00 (9.75)	0.175

**Table 5:** Comparison of three indexes between MCI group and NC group.

Group	Test durations		Lengths of gaze trajectory		Correlation degree		Drift rate	
	*M* (quartile)	*p* value	*M* (quartile)	*p* value	Mean ± SD	*p* value	*M* (quartile)	*p* value
MCI group	50.44 (85.89)	0.407	11562.63 (9539.73)	0.274	0.74 ± 0.19	0.01	0.28 (0.27)	0.019
NC group	43.39 (42.22)		10474.51 (6638.62)		0.87 ± 0.08		0.16 (0.18)	

## Data Availability

The datasets used in the experiments and discussed in the paper are available from the corresponding author on reasonable request.
